# Plant probiotic bacteria: solutions to feed the world

**DOI:** 10.3934/microbiol.2017.3.502

**Published:** 2017-06-21

**Authors:** Esther Menendez, Paula Garcia-Fraile

**Affiliations:** 1 Instituto de Ciências Agrárias e Ambientais Mediterrânicas (ICAAM), Universidade de Évora, Évora, Portugal; 2 Laboratory of Fungal Genetics and Metabolism, Institute of Microbiology, Academy of Sciences of the Czech Republic, Prague, Czech Republic

**Keywords:** plant growth promotion, biofertilizer, sustainable agriculture, beneficial bacteria

## Abstract

The increasing human population expected in the next decades, the growing demand of livestock products—which production requires higher amounts of feed products fabrication, the collective concern about food quality in industrialized countries together with the need to protect the fertility of soils, in particular, and the environment, in general, constitute as a whole big challenge that worldwide agriculture has to face nowadays. Some soil bacteria harbor mechanisms to promote plant growth, which include phytostimulation, nutrient mobilization, biocontrol of plant pathogens and abiotic stresses protection. These bacteria have also been proved as promoters of vegetable food quality. Therefore, these microbes, also so-called Plant Probiotic Bacteria, applied as biofertilizers in crop production, constitute an environmental friendly manner to contribute to produce the food and feed needed to sustain world population. In this review, we summarize some of the best-known mechanisms of plant probiotic bacteria to improve plant growth and develop a more sustainable agriculture.

## 1. Plant Probiotic Bacteria: Why Are They Necessary?

Nowadays, there is a global scenario of lack of resources to feed an ever-growing worldwide human population. Agriculture is the main primary sector involved in food production and should overcome the problem, producing sufficient nutriment for the global population. However, the reality is not as expected. Intensive agriculture has increased the occurrence of pests and diseases, promoting the use of pesticides. Moreover, intensive agriculture is based on the application of increased levels of chemical fertilizers. Both pesticides and chemical fertilizers, when used indiscriminately, can affect human and livestock health and accumulate in soils and water, polluting ecosystems. Intensive agriculture has more associated problems, i.e. reduction of the diversification of croplands, shortage of soil nutrients, loss of genetic diversity, contribution to global warming, etc. These problems become more evident in Asia and Africa, were overpopulation is a serious issue [1].

According to the Population Division of the United Nations, the world human population is estimated to reach around 9.5 billion people in 2050, and this fact will be accompanied by significant shifts in diets in developing countries, including more intake of animal origin calories, which should be satisfied by more intensive animal agriculture that will also demand more food consumption in a short period in time [2],[3]. This situation inevitably implies a search for more efficient procedures to produce animal feed and food for humans, with higher yields and more resistance to biotic and abiotic stresses [4],[5], while protecting animal and human health and, at the same time, being friendly with the environment.

On the other hand, high-quality food demand is also increasing in both developed and developing countries [6].

In this sense, the application of microorganisms, especially bacteria, with plant growth promoting features, the so-called plant probiotics, may be a possible solution to increase crop production while avoiding the above mentioned problems related to the application of chemical fertilizers and pesticides and, moreover, allowing the obtention of better quality products [6],[7].

The term Plant Probiotic Bacteria (PPB) was first mentioned by Haas and Keel [8] to name a group of microorganisms benefiting plants, which fulfils three essential criteria that combined result in better plant protection: (i) effectiveness and competitiveness in niche colonization, (ii) the ability to create induced systemic resistance (ISR) in their hosts and (iii) presence of direct antagonistic traits on pathogens. Subsets of this PPB are the ones that can be found in soils and rhizosphere, which are referred as Plant growth-promoting rhizobacteria (PGPR), term that had been proponed before by Kloepper and Schrot [9]. PGPR are naturally occurring soil bacteria, which have the ability to benefit plants in several ways, inducing the improvement of their productivity and immunity. These soil bacteria are present in the rhizosphere, in which plant roots and their exudates exert a great influence in the living being relationships occurring in this part of the soil.

PPB can be classified according to their interactions with the host plant, being divided into 2 groups: (i) free-living rhizobacteria, which live outside plant cells and enhance plant growth as a result of the metabolites that they release in the rhizosphere, and (ii) endophytes, which live inside plant tissues and/or cells and directly exchange metabolites with their host plant, positively affecting their growth [10],[11]. Most endophytic bacteria live in the intercellular spaces of the host plant; however, there are some bacteria able to form truly mutualistic interactions with their hosts and penetrate plant cell inside. Moreover, some of them are able to integrate their physiology and even go through a process of bacterial differentiation within the plant cells, resulting in the formation of specialized structures. The best known mutualistic symbiotic bacteria are the rhizobia, which establish symbiotic associations with leguminous plants, fixing atmospheric nitrogen for the plant in a specialized root structure, commonly called root nodules [12],[13]. Other examples of mutualistic bacteria associated with plants are *Frankia*, which induces the formation of nodules in actinorrhizic plants, such as *Alnus* trees, where bacterial nitrogen fixation takes place [14] or the symbiosis between cyanobacteria and cycads, amongst others [15].

A plethora of studies showed the worldwide use of PPB or PGPR as biofertilizers, contributing to the increase of crop yields and to the improvement of soil fertility; thus, these bacteria have the potential to contribute to more sustainable agriculture and forestry [16]–[19]. Bacterial biofertilizers are products in which formulation one or several bacteria that improve the nutrient status of the plants (i.e. plant growth and yield) are contained. These bacteria can benefit plant nutrient uptake through three mechanisms: (i) replace soil nutrients and/or (ii) make nutrients available to plants and/or (iii) increase plant access to those nutrients [20]. Nonetheless, these plant growth-promoting bacteria may have other mechanisms to promote plant growth, such as phytohormone biosynthesis, mechanisms to reduce or avoid environmental stresses and/or the prevention of plant diseases induced by pathogens.

In this review, we summarize the main mechanisms of plant growth promotion presented by bacterial species, which were reported as able to improve crops yields and hence refer to updated studies that have evaluated the potential applications of a wide diversity of bacterial isolates in different plants, mostly with agronomical interest. Finally, we will discuss some aspects of the current application of those strains as biofertilizers and suggest future perspectives concerning the role of bacterial-based biofertilizers in sustainable agro-ecosystems.

## 2. Plant Growth Promoting Mechanisms

Rhizobacteria can promote plant growth through a broad range of mechanisms, which can be grouped according to their mode of action in: (i) the synthesis of substances that can be assimilated directly by plants, (ii) the mobilization of nutrients, (iii) the induction of plant stress resistance, (iv) the prevention of plant diseases. PGPR presenting one or several of those plant growth-promoting traits are summarized in Table 1.

**Table 1.** Plant growth-promoting mechanims exhibited by Plant Probiotic Bacteria in several crops.

Plant growth promotion (PGP) traits	PGP Rhizobacteria (genus level)	Crop type	References
Nitrogen fixation	*Azoarcus*	rice	[21]
	*Azorhizobium*	wheat	[22]
	*Azospirillum*	several cereals, sugarcane, bean, soybean	[23]–[27]
	*Azotobacter*	several cereals, lineseed, tobacco, sunflower, tea, coffee, coconut tree, beetroot, tomato	[28],[29],[30]
	*Bacillus*	rice	[31],[32]
	*Brevundimonas*	wheat	[33]
	*Burkholderia*	rice	[34],[35]
	Enterobacteriales	maize, wheat, sugarcane	[33],[36],[37]
	*Frankia*	*Alnus*	[38]
	*Gluconacetobacter*	sugarcane	[39]
	*Herbaspirillum*	sugarcane, bean, rice, sorghum, maize	[40],[41],[42]
	*Paenibacillus*	rice, canola	[31],[43]
	*Pseudomonas*	rice	[32]
	*Rhizobium* and related genera	leguminous plants	[44]

Phytohormone biosynthesis (auxins, gibberelings, cytokynins, ethylene and ACC desaminase synthesis)	*Azobacter*	cucumber	[45]
	*Bacillus*	potato, cucumber, oriental thuja, pepper, rice	[33],[46]–[49]
	Enterobacteriales	sugarcane, wheat, pepper, soybean	[33],[37],[50],[51]
	*Hartmannibacter*	summer barley	[52]
	*Paenibacillus*	lodgepole pine, rice, barley, wheat	[31],[53],[54]
	*Phyllobacterium*	*Arabidopsis*	[7],[55]
	*Pseudomonas*	wheat, mung bean	[56],[57]
	*Rhizobium* and related genera	pepper, tomato, lettuce, carrot, strawberries, carnation, chickpea, mung bean, hop clover	[6],[57]–[63]
	*Sphinogomonas*	tomato, soybean	[51],[64]
	*Streptomyces*	indian lilac, cocoa	[65],[66]

Phosphate solubilization	*Bacillus*	rice	[31]
	*Burkholderia*	rice	[67]
	Enterobacteriales	wheat	[33]
	*Herbaspirillum*	rice	[67]
	*Paenibacillus*	rice	[31]
	*Phyllobacterium*	strawberries	[7]
	*Rhizobium* and related genera	pepper, tomato, lettuce, carrot, strawberries, carnation, chickpea	[6],[58],[60],[61],[62]
	*Streptomyces*	wheat	[68]

Potassium solubilization	*Bacillus*	wheat, maize Sudan grass, eggplants, pepper, cucumber, cotton, rape, groundnut	[69]–[74]
	Enterobacteriales	tobacco	[74],[75]
	*Frauteria*	tobacco	[76]
	*Microbacterium*	tobacco	[75]
	*Paenibacillus*	black pepper	[74]
	*Pseudomonas*	tobacco, tea	[77]

Siderophore production	*Bacillus*	maize, pepper, rice	[31],[78]
	*Chryseobacterium*	tomato	[79]
	Enterobacteriales	wheat	[33]
	*Micrococcus*	maize, canola	[80]
	*Phyllobacterium*	strawberries	[7]
	*Pseudomonas*	potato, maize	[78]
	*Rhizobium* and related genera	pepper, tomato, lettuce, carrot, strawberries, carnation, chickpea	[6],[58],[60],[61],[62]
	*Stenotrophomonas*	maize, canola	[80]
	*Streptomyces*	indian lilac, cocoa	[65],[66]

Biocontrolers (production of plant cell wall degrading enzymes, induced disease suppression, resistance to stresses…)	*Bacillus*	maize, peanut, Chinese cabbage, cucumber, tomato, lettuce, banana, berries, pepper, cucumber, mint	[81]–[84]
	Enterobacteriales	tomato, wheat, apple tree	[33],[85],[86],[87]
	*Mycobacterium*	maize	[81]
	*Paenibacillus*	tomato, pepper, barley, wheat	[54],[88],[89]
	*Pseudomonas*	cotton, maize, pidgeon pea, wheat, rice, cucumber, tomato	[31],[81],[84],[90],[91],[92]
	*Rhizobium* and related genera	peanut, pidgeon pea	[82],[91]
	*Streptomyces*	cocoa, wheat	[66],[68]

### 2.1. Interesting molecules and substances assimilation and/or biosynthesis

#### 2.1.1. PPB and nitrogen fixation

Although is quite abundant in the Earth, nitrogen (N) is the most limiting nutrient for plants, whose require it for the formation of aminoacids and subsequently, proteins. Some prokaryotes have the exclusive of managing the process of combination or conversion of atmospheric nitrogen into organic forms, which can be finally assimilated by plants [93]. Amongst free-living rhizobacteria, members of the genera *Azospirillum, Azotobacter, Beijerinckia, Bacillus, Paenibacillus, Burkholderia, Gluconoacetobacter* and *Herbaspirillum* were reported as nitrogen-fixing microorganisms. The genus *Azospirillum* is commonly associated with cereals in temperate zones, increasing crop yields in most of them, as well as in some legumes and sugarcane [23]–[27]. Members of the genus *Azotobacter* are able to fix nitrogen in rice crops [30]. Moreover, some species of this genus are tested as biofertilizers for several cereals, such as wheat, barley, oat, rice or maize; oil plants, such as, linseeds and sunflowers; and other variety of plants, such as beetroot, tobacco, tea, coffee and coconuts (reviewed in [29]).

Some species belonging to the genera *Gluconacetobacter*, *Azospirillum* and *Herbaspirillum* are frequent sugarcane endophytes and act as nitrogen fixers contributing to this plant nutrition [23],[39],[40]. The genus *Herbaspirillum* has also been identified as a nitrogen fixing endophyte of several crops [40],[41],[42]. Last but not least, the genera *Bacillus* and *Paenibacillus* are free-living nitrogen fixers and have other PGP traits, which make them suitable candidates for application [94],[95].

Moreover, some of those free-living bacteria may enter roots of some crops, such as species of *Azoarcus*, *Azospirillum* and *Burkholderia* in rice roots, which increase the nitrogen concentration of this specific crop [21],[35],[96],[97] or nitrogen-fixing *Azorhizobium* strains in wheat plants [22]. Interestingly, some strains of the genera *Rhizobium* and *Bradyrhizobium*, which were found in association with rice and wheat roots, increase this nutrient concentration in those plant yields [98],[99],[100].

On the other hand, certain diazotrophic bacteria are able to establish truly mutualistic symbiosis within plant tissues, mainly through the formation of root nodules. These symbioses are found between rhizobia and legumes, rhizobia and *Parasponia*, *Frankia* and actinorhizal plants and cyanobacteria and cycads [15],[38],[44],[101],[102],[103].

#### 2.1.2. PPB and phytohormone biosynthesis

Many bacterial endophytes are able to synthetize phytohormones, which are defined as organic molecules involved in several processes of the different stages of plant growth and development. The biosynthesis of these phytohormones by certain microorganisms might be involved in plant pathogenesis; however, a wide spectrum of beneficial bacteria are able to produce them and have them involved in plant growth and development as plant growth promotion traits [104],[105].

Amongst the phytohormone-producing PGP rhizobacteria, we will focus in the ones producing auxins, cytokinins, gibberellins and ethylene. Each one of these phytohormones are involved in key processes of plant development [106].

Auxins are phytohormones produced by several bacteria, being these compounds key signalling molecules for bacterial communication in order to coordinate activities. Amongst these auxins, indole-3-acetic acid (IAA) is the best known and most active auxin in plants. Cytokinins promote cytokinesis, vascular cambium sensitivity, vascular differentiation and root apical dominance. Gibberellins are involved in seed germination and emergence, stem and leaf growth, floral induction and flower and fruit development. In last place, ethylene is a plant hormone known to regulate several processes such as fruit ripening, flower blooming or leaves abscission. However, it also promotes seed germination, secondary root formation and root-hair elongation. All of these phytohormones are present in PGP rhizobacteria [107]–[112].

Auxin-producing *Bacillus* spp. have been reported to exert a positive effect in the development of several crops, such as *Solanun tuberosum* (potato) or *Oryza sativa* (rice) [31],[46],[47]. Moreover, members of the genus *Bacillus* were reported as cytokinin producers [47],[48],[49]. *Bacillus* *megaterium* and also *Azotobacter* *chroococcum* strains were found to produce cytokinins and promote cucumber growth [45]. Liu et al. [48] reported that oriental thuja seedlings inoculated with cytokinin-producing *Bacillus* *subtilis* strains have better resistance to drought stress. Moreover, a gibberellins-producing strain of *Bacillus* *cereus* enhances the growth of red pepper plants [113].

The genus *Paenibacillus* was also reported as a good phytohormone producer. Bent et al. [53] have reported elevated root IAA level in lodgepole pine (*Pinus contorta*) plantlets inoculated with a strain of *Paenibacillus* *polymyxa*. Moreover, other studies report the effects of the genus *Paenibacillus* as phytohormone producer for rice, barley and wheat plant crops [31],[54].

*Enterobacter* and related Enterobacteriales are also good phytohormone producers and have PGP effects in sugarcane, wheat, pepper and soybean, amongst others [33],[37],[50],[51],[114].

Interestingly, rhizobia are also described as phytohormone synthesizers. IAA-producing rhizobial strains improve the growth of several crops, such as *Capsicum* *annuum* (pepper), *Solanum lycopersicum* (tomato), *Fragaria anannasa* (strawberry), *Dianthus caryophyllus* (red carnation), *Lactuca* *sativa* (lettuce) and *Daucus* *carota* (carrot) [6],[57]–[63]. Moreover, *Rhizobium* *leguminosarum* strains isolated from Delta Nile rice fields in rotation with clover were reported as producers of auxins and gibberellins, amongst other phytohormones [99],[100].

The genus *Sphingomonas* was also reported as phytohormone-producing bacteria; tomato plants inoculated with the gibberellin-producing *Sphingomonas* sp. LK11 strain showed a significant increment in several growth attributes [63]. Moreover, in a recent study, Asaf et al. [51] reported the positive effect and the production of phytohormones of *Sphingomonas* and *Serratia*, an enterobacteria, in soybean plant development.

In case of actinobacteria, there are some studies reported that endophytic *Streptomyces* strains produce IAA and are potential plant growth promoters in *Azadirachta indica* (indian lilac) and cocoa [65],[66].

Some bacteria produce ACC deaminase (1-aminocyclopropane-1-carboxylate deaminase), to hydrolyze the ethylene precursor in plants (ACC), to obtain ammonia and α-ketobutyrate, which can be used as a source nitrogen and carbon. Therefore, these bacteria modulate ethylene levels in plants and hence, prevent some of the negative effects produced by high ethylene concentrations [115]–[119]. Moreover, these molecules have an important role in the nodulation process between a rhizobial strain and a legume (reviewed in [120]). Amongst rhizobia, there are many members presenting ACC deaminase production. *Rhizobium* *leguminosarum* strains producing ACC-deaminase promoted pepper and tomato plant growth [6]. *Ensifer meliloti* strain expressing an exogenous *acdS* gene (ACC deaminase gene) enhances plant development in *Medicago lupulina* (hop clover) [63].

Moreover, the exogenous expression of an *acdS* gene in a *Mesorhizobium* strain improved chickpea plants growth under salt stress [59]. It is also shown that other members of rhizobia, such as *Phyllobacterium* genus, are able to produce this compound. For example, strain STM196 of *Phyllobacterium* *brassicacearum* emits ethylene and contributes to root hair elongation in *Arabidopsis* *thaliana* [55]. Also, β-rhizobia members are able to synthesize ACC deaminase (reviewed in [120]).

Although a high number of rhizobial and related species are able to produce ACC deaminase, the genus *Pseudomonas* is the first producer of this particular molecule [118]. Indeed, ACC deaminase was first purified from a *Pseudomonas* strain [121]. Shaharoona et al. [56] reported that two ACC-deaminase-containing *Pseudomonas* strains improved the growth and yield of wheat crops, with varying levels of NPK nutrients. Magnucka and Pietr [122] reported that various strains of ACC-producing *Pseudomonas* benefit the growth of wheat seedlings. Zerrouk et al. [123] showed that a *Pseudomonas* strain isolated from date palm rhizosphere, was able to improve the development of maize plants under two different stresses, as revealed by the increase in all parameters measured under both salt and aluminum stress.

Moreover, a combination of *Rhizobium* and *Pseudomonas* ACC-deaminase-producing strains improve the growth, physiology and quality of mung beans under saline stress conditions and of *Pisum* *sativum* (pea) cultivated on alluvial soils [57] and a combination of *Serratia* and *Pseudomonas* ACC-deaminase-producing strains improved the yield of wheat plants in saline conditions [124].

Suarez et al. [52] described an ACC-deaminase producing strain of *Hartmannibacter diazotrophicus*, which act as PGPR increasing plant growth in barley (*Hordeum* *vulgare*) in saline soils.

### 2.2. PPB and nutrient mobilization

#### 2.2.1. Phosphorous solubilizers (PSB)

Phosphorous (P) is the second essential nutrient for plants, after nitrogen (N) and the major part of the reservoirs are not available for them. This element is quite insoluble in soils and accordingly, this element was applied exogenously in traditional agriculture as chemical P fertilizers. Nevertheless, when applied as fertilizer to crop fields, P passes rapidly to become insoluble and thus, unavailable to plants [125],[126].

Therefore, the use of P-solubilizing bacteria (PSB) might represent a green substitute for these environment-damaging chemical P fertilizers. Soil bacteria such as the genera *Micrococcus, Pseudomonas, Bacillus, Paenibacillus, Deftia, Azotobacter, Klebsiella, Pantoea* and *Flavobacterium*, amongst others, have been reported to be efficient phosphate solubilizers [127],[128],[129]. Moreover, there are many phosphate-solubilizing rhizobial strains, which promote the growth of several crops, such as *Daucus carota, Lactuca sativa*, strawberries, ornamental plants and legumes, amongst other crops of economic interest [58],[60],[61] and a *Phyllobacterium* strain able to solubilize phosphates improves the quality of strawberries [7]. Garcia-Fraile et al. [6] reported two *Rhizobium leguminosarum* strains that solubilize phosphate and are proper PGPR for pepper and tomato plants. The genus *Mesorhizobium* has also strains that are good P-solubilizers, promoting the growth of chickpea and barley [59],[130].

Liu et al. [131] isolated several PSB strains from betel nut (*Areca catechu*), a slender palm growing in tropical regions, which improve its host growth. These PSB strains belong to different genera, such as *Bacillus, Paenibacillus, Shigella, Enterobacter, Escherichia, Acinetobacter, Kurthia* and *Rhizobium*. Also in tropical soils, a strain of *Burkholderia*, a β-rhizobia member, was reported as PSB for *Lycopodium cernuum* plants.

In a screening for PSB, Panda et al. [132] isolated strains belonging to the genera *Bacillus, Pseudomonas, Micrococcus, Staphylococcus, Microbacterium* and *Delftia* from various crops and demonstrated that these strains also have antagonistic properties. *Streptomyces* spp. were also described as PSB for wheat plants [68].

#### 2.2.2. Potassium solubilizers (KSB)

After nitrogen and phosphorous, potassium (K) is the third nutrient essential for plant growth. Some rhizobacteria are able to make available the insoluble potassium forms [133]. There is quite a diversity of K-solubilizing bacterial genera (KSB) [74]. Amongst Firmicutes, there are many examples of KSB. The genera *Bacillus* and *Paenibacillus* are one of the most reported KSB. *Bacillus edaphicus* has been reported to increase potassium uptake in wheat [134] and *Paenibacillus glucanolyticus* was found to increase the dry weight of black pepper [135]. Sudan grass inoculated with the potassium-solubilizing bacterium *Bacillus mucilaginosus* had higher biomass yields [136]. Wheat and maize plants inoculated with the KSB *Bacillus mucilaginosus* under laboratory controlled conditions showed increased plant biomass and chlorophyll content in leaves [73]. Also, *Bacillus mucilaginosus* in coinoculation with the phosphate-solubilizing *Bacillus megaterium* promoted the growth of groundnut, eggplant, pepper and cucumber [70],[71],[72]. Amongst Proteobacteria, the genus *Pseudomonas* was described as KSB, benefiting growth and development of tea plants (*Camellia* *sinensis*) [77] and tobacco [75]. More recently, a strain of the genus *Frauteria* was described as KSB for tobacco [76]. Zhang and Kong [75] also found a KSB strain belonging to the genus *Microbacterium* (*M. foliorum*), an Actinobacteria, which has positive effect on tobacco plants.

#### 2.2.3. Siderophore production in PPB

Siderophores are organic compounds with the main function to kidnap the ferric iron (Fe^3+^) from the environment [137]. In the cases when Fe^3+^ is a limiting nutrient to the plants, siderophores from soil microorganisms fix this problem. However, the accurate mechanisms of how plants are supplied with Fe by these microbes supply are not well understood yet [138],[139]. Siderophores from endophytic *Streptomyces* strains are able to promote *Azadirachta* *indica* and *Theobroma cacao* plant growth [64],[65]. Rhizobial strains able to produce siderophores have been reported as potential biofertilizers, improving the production of carrot, lettuce, pepper, tomato, strawberry, red carnation and chickpea [6],[58],[50]–[62]. Moreover, the strain PEPV15 of *Phyllobacterium* *endophyticum*, a siderophore-producing strain, promotes the growth and quality of strawberries [7]. Ghavami et al. [80] isolated several strains belonging to the genera *Micrococcus* and *Stenotrophomonas* from the rhizosphere of *Brassica napus* (canola). Some of these strains produce siderophores, which contributed to the improvement of maize and canola plant growth under greenhouse conditions. Siderophores produced by *Chryseobacterium* sp. C138 are effective in supplying Fe to iron-starved tomato plants [79].

### 2.3. PPB as biocontrolers and plant protectors

#### 2.3.1. PPB conferring resistance to stresses

Abiotic stress in plants, originated in situations such as drought, flooding conditions, extreme temperatures or salinity, heavy metal-produced phytotoxicity and oxidative stress, are the primary cause of crop loss worldwide [140],[141].

Liddycoat et al. [142] described *Pseudomonas* strains that enhance asparagus seed germination and seedling growth under water-stress conditions generated in greenhouse conditions. Moreover, *P. fluorescens* strain MSP-393 acts as a PGPR for several crops grown in the saline soils of coastal ecosystems and *P.* *putida* Rs-198 promotes cotton seedlings growth (increases germination rate) under saline stress. These *Pseudomonas* species act as protectors against salt stress, increasing Mg^2+^, K^+^ and Ca^2+^ absorption, decreasing Na^+^ uptake and improving endogenous IAA production [80],[143]. The inoculation of maize plants cultivated in salt-stress conditions with rhizobial strains showed a similar efficiency to the showed by the application of N fertilizer in the same crop [81]. El-Akhal et al. [82] described that strains of *Paenibacillus alcaligenes, Bacillus polymyxa* and *Mycobacterium* *phlei* are able to improve *Arachis* *hypogaea* growth and nutrient uptake under high temperature conditions as well as under salinity.

Yaish et al. [144] isolated many diverse rhizobacterial genera from date palm plantlets, which most of them present various PGPR traits, contributing to plant growth and development in conditions of high degree of salinity.

Rhizobacteria with PGPR mechanisms are also phytoremediators, which have the ability of degrading pollutants, allowing plant development in contaminated soils (reviewed in [141],[145],[146]). Species of the genera *Sphingomonas* and *Microbacterium* make plants of *Alyssum* *murale* to uptake Ni from contaminated soils [147]. Moreover, Ni can be accumulated by *Brassica* *juncea* plants inoculated with *Pseudomonas* and *Bacillus* strains, which produced siderophores, ACC-deaminase and phytohormones [148]. In a similar way, Dimkpa et al. [149] described that the siderophores produced by a strain of *Streptomyces* *acidiscabies* bind Ni, helping to cowpea (*Vigna* *unguiculata*) plants to develop in nickel-contaminated soils. The genera *Pseudomonas*, *Azospirillum* and *Rhizobium*, apart from their well know PGPR potential, were also reported as protectors of *Medicago sativa* seeds in copper-contaminated soils [150].

#### 2.3.2. PPB and prevention of plant diseases

The mechanisms of bacterial plant disease prevention may be direct or indirect, depending on if pathogens are inhibited as a result from PGPR metabolism or the PGPR strains compete with pathogens. The production of antibiotics, siderophores and cell wall degrading enzymes are mechanisms that can be included in this section [78],[151].

Some PGPR synthesize antibiotic substances that inhibit the growth of some plant pathogens [152],[153]. For instance, *Pseudomonas* spp. produces antibiotics that inhibit *Gaeumannomyces graminis* var. tritici, the causal agent of take-all (white heads) of wheat [154]. Moreover, different species of the genera *Klebsiella*, *Bacillus*, *Acinetobacter* and *Paenibacillus*, which were resistant to high concentrations of selenium (Se), acted as biocontrolers of the same pathogen of wheat [155].

A high number of strains belonging to *Bacillus* spp. are able to produce antibiotics that are active against Gram-positive and Gram-negative bacteria, as well as many pathogenic fungi [156]. *Bacillus* *cereus* UW85 contributes to the biocontrol of alfalfa damping-off [157]. Two strains of *Bacillus* *subtilis* are able to produce antibiotics against several pathogens affecting soybean seeds, as well as enhance the development of this plant [158].

Plant cell wall hydrolytic enzymes (cellulases, chitinases and β-glucanases) are also involved in the biocontrol of pathogens, mostly fungi, since cellulose, chitin and β-glucan are the major fungal cell wall components. Bacteria producing chitinases and β-glucanases inhibit fungal growth. Kumar et al. [91] have reported that *Sinorhizobium fredii* KCC5 and *Pseudomonas fluorescens* LPK2 produce chitinases and β-glucanases and control the disease produced by *Fusarium* *udum*. Other *Pseudomonas* spp., which exhibits chitinases and β-glucanases production, inhibits the infection of *Rhizoctonia* *solani* and *Phytophthora* *capsici*, two of the most destructive crop pathogens in the world [159]. A combination of a cellulase-producing *Micromonospora* and an antibiotic-producing *Streptomyces* species were shown to suppress root rot of *Banksia* *grandis* plants caused by the pathogen *Phytophthora* *cinnamomi* [160]. Several cellulase-producing actinobacterial genera were reported as biocontrolers of the damping-off disease of cucumber plants, caused by *Pythium* *aphanidermatum* [161].

The genus *Micromonospora*, actinobacterial genus also reported as plant probiotic [162], is able to induce systemic resistance in tomato plants affected by *Botrytis* *cinerea* [163]. This genus was described a high producer of hydrolytic enzymes [164].

Some rhizobacteria are able to synthesize proteins with toxic properties against certain crop insect pests. For example, *Bacillus* *thuringiensis* subsp. *kurstaki* HD-1 has been widely used in the forest industry for controlling the gypsy moth [165]. Also, bacteria belonging to the genera *Photorhabdus* and *Xenorhabdus*, which are enterobacteria associated with entomopathogenic nematodes, inhibit harmful insects, existing nematodal-bacterial formulations for controlling insect populations in the fields [166]. Bano and Muqarab [167] reported that a biopesticide formulated with PGPR strains of *Pseudomonas* *putida* and *Rothia* sp. induced plant defence responses against *Spodoptera litura*, a worm affecting tomato plants. In a recent study, a combination of two *Pseudomonas* species and a specie of AMF protect tomato plants against root-knot nematodes [168].

Microbial siderophores, apart from being a PGP trait itself, are also involved in the control of plant pathogens by limiting the Fe available for the phytopathogens [78],[146]. In this sense, *Pseudomonas* siderophores control *Fuxarium* *oxysporum* infection in potato plants [169]. *Pseudomonas* and *Bacillus* strains produce siderophores that inhibit several fungal pathogens in maize plants [170]. Yu et al. [171] reported that a siderophore-producing strain, which is identified as *Bacillus* *subtilis*, exerts a biological control effect on *Fusarium* wilt and promotes pepper growth. Verma et al. [65] reported that endophytic *Streptomyces* strains isolated from Indian lilac (*Azadirachta* *indica*) produce siderophores with biocontrol potential.

Bacterial species producing phytohormones are also proper biocontrolers for phytoplasm-induced diseases. Gamalero et al. [172] reported how an ACC deaminase-producing *Pseudomonas* strain help the plant to reduce the stress generated by the infection of the flavescence doreé phytoplasms.

Some members of Enterobacteriales are able to increase the induced systemic response to pathogens and ameliorate stress related to environmental conditions, such as salinity. For example, a strain of *Serratia* *marcescens* showing PGPR traits benefit salt-stressed wheat plants [86].

In general terms, *Bacillus* and *Paenibacillus* reveal themselves as proper biocontrolers and PGPR [173]. Senthilkumar et al. [174] described the use of various strains of these genera against the charcoal rot disease in soybean, caused by the fungus *Rhizoctonia* *bataticola*. Herrera et al. [175] reported the isolation of *Paenibacillus* spp. and *Pantoea* spp. strains from wheat seeds, which showed PGPR traits and were efficient biocontrolers alone and in combination against the fungus *Fusarium* *graminearum* in wheat and barley kernels. Andreolli et al. [176] isolated a diversity of bacterial endophytes from grapevines of different ages, showing that *Bacillus* spp. were the best PGPR and biocontrolers with activity against *Botrytis cinerea* and other fungi.

## 3. Conclusions and Future Perspectives of the Application of PPB for a Sustainable Agriculture

Nowadays, worldwide agriculture faces several challenges: (i) enough sustainable food and feed production to satisfy the increasing demand of a raising human population with an expanding demand of livestock products, (ii) using limited resources (i.e. fertile soils), (iii) caring about environmental problems induced by traditional practises of intensive farming and (iv) fulfilling the food quality requirements of the markets in developed countries. In a great effort to summarize all the issues related to traditional agriculture, Pretty et al. [177] published a list of 100 questions that must be taken into account for a future worldwide agriculture. Some questions described in the first section of this manuscript deal with the application of organic fertilizers (biofertilizers) to improve food productivity and the authors suggest that native soil organisms can be exploited for that purpose.

In this review, we summarized a plethora of studies recently published, which show the potential of plant probiotic bacteria to produce benefits for plants in several ways. These benefits include phytostimulation, nutrient mobilization and biocontrol of plant pathogens. Moreover, this group of beneficial bacteria might help alleviating stresses produced by several factors, such as salinity or heavy-metal accumulation, amongst others. These bacteria have also been proved as promoters of vegetable food quality.

Therefore, it seems that plant probiotic bacteria, applied as biofertilizers formulated with single strains or with a consortia of isolates combining different beneficial effects, could serve as a possible solution to feed the world while protecting ecosystems and improving food quality. Consequently, the establishment of a dialogue among scientists, politicians and farmers as well as the existence of research programs and policies should be occurring oftenly in order to join efforts for the development effective and safe products based of PPB, which will bring benefits not just for producers, but for the whole human being as well as for the entire Planet.
